# The evaluation of copy number variants in an unselected population of patients with inherited cardiac conditions: the INTERACTION study

**DOI:** 10.1093/europace/euag150

**Published:** 2026-06-18

**Authors:** Giulia Corona, Simona Mellone, Monia Magliozzi, Maria Gnazzo, Martina Manzoni, Carmela Fusco, Silvia Morlino, Italia Loddo, Floriana Barbera, Luigi Alioto, Stefania Paganini, Luisa Cucugliato, Andrea Faggiano, Valentina Andrioletti, Rossana Mineri, Francesca Girolami, Ivan Limongelli, Mara Giordano, Valeria Novelli

**Affiliations:** Centro Cardiologico Monzino IRCCS, Unit of Cardiovascular Genetics, 20138 Milan, Italy; Laboratory of Medical Genetics, S.C.D.U. Clinical Biochemistry, A.O.U. ‘Maggiore Della Carità’, Novara, Italy; Laboratory of Medical Genetics, Translational Cytogenomics Research Unit, Bambino Gesù Children Hospital, IRCCS, Rome, Italy; Laboratory of Medical Genetics, Translational Cytogenomics Research Unit, Bambino Gesù Children Hospital, IRCCS, Rome, Italy; Centro Cardiologico Monzino IRCCS, Unit of Cardiovascular Genetics, 20138 Milan, Italy; Division of Medical Genetics, Fondazione IRCCS-Casa Sollievo Della Sofferenza, San Giovanni Rotondo, Italy; Division of Medical Genetics, Fondazione IRCCS-Casa Sollievo Della Sofferenza, San Giovanni Rotondo, Italy; Department of Diagnostic and Therapeutic Services, Pathology Unit, Istituto di Ricovero e Cura a Carattere Scientifico-Istituto Mediterraneo per I Trapianti e Terapie ad Alta Specializzazione (IRCCS-ISMETT), Palermo 90127, Italy; Department of Diagnostic and Therapeutic Services, Pathology Unit, Istituto di Ricovero e Cura a Carattere Scientifico-Istituto Mediterraneo per I Trapianti e Terapie ad Alta Specializzazione (IRCCS-ISMETT), Palermo 90127, Italy; Department of Diagnostic and Therapeutic Services, Pathology Unit, Istituto di Ricovero e Cura a Carattere Scientifico-Istituto Mediterraneo per I Trapianti e Terapie ad Alta Specializzazione (IRCCS-ISMETT), Palermo 90127, Italy; Department of Cardio-Thoracic-Vascular Diseases, Foundation IRCCS Ca’ Granda Ospedale Maggiore Policlinico, Milan 20122, Italy; enGenome Srl, Pavia, Italy; Department of Cardio-Thoracic-Vascular Diseases, Foundation IRCCS Ca’ Granda Ospedale Maggiore Policlinico, Milan 20122, Italy; enGenome Srl, Pavia, Italy; Unit of Medical Genetics, IRCCS Humanitas Research Hospital, Via Manzoni 56, Rozzano, MI, Italy; Cardiology Unit, Meyer Children’s Hospital IRCCS, Florence, Italy; enGenome Srl, Pavia, Italy; Laboratory of Medical Genetics, S.C.D.U. Clinical Biochemistry, A.O.U. ‘Maggiore Della Carità’, Novara, Italy; Department of Health Sciences, Università del Piemonte Orientale, 28100 Novara, Italy; Centro Cardiologico Monzino IRCCS, Unit of Cardiovascular Genetics, 20138 Milan, Italy

**Keywords:** Copy Number Variants, Inherited Cardiac Conditions, Genetic Testing Strategy

## Abstract

**Aims:**

The current diagnostic approach to inherited cardiac conditions (ICCs) is primarily focused on the analysis of single-nucleotide variants (SNVs) and small insertions/deletions (InDels). However, as recommended for other inherited diseases, the analysis of Copy Number Variants (CNVs) should be equally considered. In cardiology, the diagnostic contribution of CNVs remains insufficiently studied, with limited evidence and no standardized recommendations for analytical workflows. This study aims to assess the prevalence of pathogenic CNVs in an Italian multicentre cohort of ICC patients, providing recommendations for integrating CNV analysis into routine genetic testing.

**Methods and results:**

A total of 203 ICC probands with prior negative or inconclusive results for SNVs and InDels testing were included. The tertiary bioinformatic analysis was performed by eVai enGenome software, and putative CNVs were validated by Multiplex Ligation-dependent Probe Amplification (MLPA) assays. MLPA confirmed only one of the five initially suspected CNVs, identifying a deletion in *MYBPC3* gene classified as pathogenic according to ACMG/AMP guidelines. This result confirmed a limited diagnostic yield of 0.50%. Considering the substantial costs, time constraints, and specialized expertise required, we propose a strategy to prioritize selected ICC patients for CNV analysis.

**Conclusion:**

Evidence from this real-world cohort suggests the incorporation of CNV analysis as a second-tier test in patients with ICC and specific clinical or molecular indications.

## Graphical Abstract

**Graphical Abstract:**
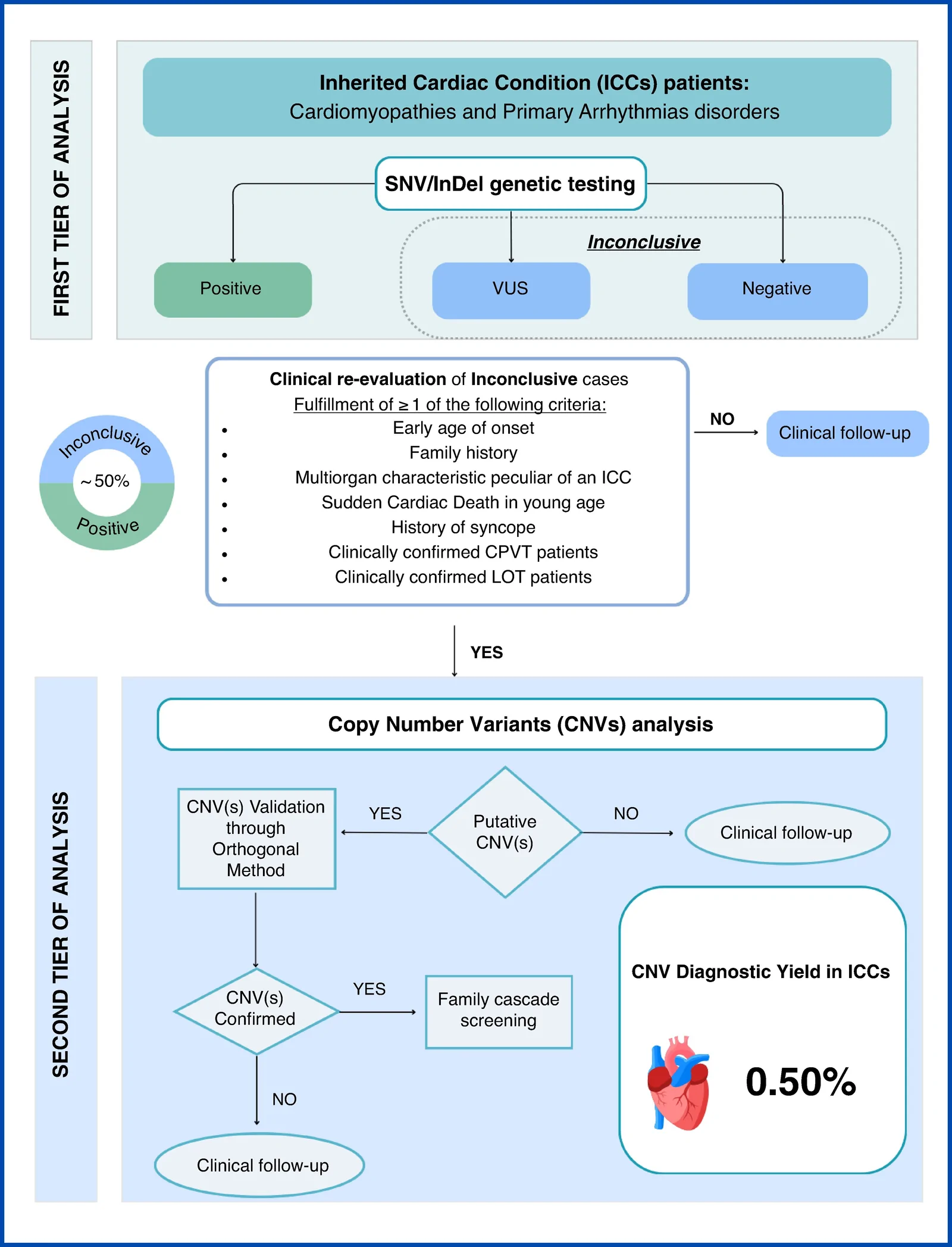

What’s New?

- Low diagnostic yield of CNV analysis in ICC patients.
- Inherited Cardiac Conditions.
- Contribution to the standardization of CNV analysis in cardiology.
- Copy Number Variants.
- Recommendation for second-tier CNV testing for high-risk patients.

## Introduction

Inherited cardiac conditions (ICCs) represent a heterogeneous group of diseases frequently associated with an increased risk of sudden cardiac death (SCD) at a young age.^1^ Over the past decade, advances in molecular diagnostics have played a crucial role in elucidating the genetic basis of inherited diseases, including cardiomyopathies and primary arrhythmogenic diseases.

Genetic testing is now considered a powerful tool to confirm the diagnosis, improve the clinical management of patients, identify the most effective therapeutic strategies, and facilitate cascade screening for early identification of family members at risk.

The current diagnostic approach for ICCs is primarily focused on the identification of single-nucleotide variants (SNVs) and small insertions/deletions (InDels) with an overall yield of ∼50%.^2^ However, this rate varies across different ICCs, ranging from around 20% for Brugada syndrome (BrS) to up to 80% for long QT syndrome (LQTS).

The absence of identifiable pathogenic variants in many ICC patients suggests that a more comprehensive genetic analysis, incorporating the evaluation of different types of genetic alterations such as variants in non-coding regions and structural variations (SVs), may enhance the sensitivity of screening.

Copy Number Variants (CNVs) are recognized as pathogenic alterations in several inherited diseases, with reported yields depending on the specific condition and the gene panels.^3^ In a large study of 143 515 unrelated individuals referred for diagnostic NGS gene panels, a high proportion of pathogenic CNVs (35% overall) was identified in panels for neurological disorders, while lower proportions were observed in panels for cardiovascular diseases (4.7% overall) and hereditary cancer syndromes (8.3% overall).^3^ Despite the well-established role of these variants—duplications or deletions from > 50 bp to more than 1 Mb^4^ reported detection rates in ICCs remain highly variable across different cohorts, highlighting the importance of further characterizing their prevalence through standardized diagnostic pipelines.

Previous studies, focused on the identification of CNVs, reported a detection rate of 0.3% to 14% among patients with SCD-related diseases.^5,6^ These differences in detection rates are attributable to several variables, including the type of gene panel adopted, the clinical and genetic criteria used for patient selection, and the methodologies applied for CNV confirmation and interpretation.

These findings highlight the potential pathogenic role of CNVs in ICC and support their integration into routine genetic testing.^2^

To further investigate this in a real-world setting, we evaluated the incidence of pathogenic and likely pathogenic CNVs in a multicentre Italian cohort of ICC patients in the absence of pathogenic or likely pathogenic SNVs or InDels in disease-causing genes.

Furthermore, we provide recommendations for the integration of CNVs analysis into the routine molecular diagnostic workflow for ICC patients.

## Methods

### Study cohort

The cohort includes 203 probands diagnosed with ICCs, referred from eight different Italian genetic testing laboratories, affiliated with Research Institutes or University Hospitals.

All probands underwent comprehensive clinical evaluation based on their cardiac phenotype, and LQTS diagnosis was established in the presence of a QTc ≥480 ms, and in the absence of acquired causes or a Schwartz score ≥3.5.

Phenotypes include Arrhythmogenic Cardiomyopathy (ACM) (*n* = 25), Familial Dilated Cardiomyopathy (DCM) (*n* = 53), Hypertrophic Cardiomyopathy (HCM) (*n* = 52), Long QT syndrome (LQTS) (*n* = 18), Brugada syndrome (BrS) (*n* = 39), Catecholaminergic Polymorphic Ventricular Tachycardia (CPVT) (*n* = 5), and a group of patients experienced Idiopathic ventricular fibrillation/Cardiac Arrest/Sudden Cardiac Death/Ventricular Tachycardia (SCD) (*n* = 11).

All patients underwent genetic testing, providing written informed consent, including an authorization for the anonymous use of their data for research purposes and/or scientific publications. Genetic testing did not identify any pathogenic or likely pathogenic SNVs or small insertions and deletions (indels).

### Sequencing approaches

Different Next Generation Sequencing (NGS) strategies were adopted: 2 laboratories (Ospedale Pediatrico Bambin Gesù di Roma and Ospedale Maggiore Policlinico di Milano) performed Exome sequencing (WES), for a total of 37 patients and 6 laboratories (Centro Cardiologico Monzino di Milano, Ospedale Maggiore della Carità di Novara, Casa Sollievo della Sofferenza di San Giovanni Rotondo, ISMETT di Palermo, Humanitas di Rozzano, Azienda Ospedaliera Universitaria Meyer di Firenze) used different custom sequencing panel for the remaining 166 patients.

A common *in silico* panel of 49 genes, including 33 genes associated with cardiomyopathies and 16 with Primary arrhythmia disorders, has been developed for the study (*Table 1*).

**Table 1:** List of genes included in the in-silico panel

**Genes associated with**	*ABCC9* (NM_005691.4)	*JPH2* (NM_020433.5)	*PRKAG2* (NM_016203.4)
**cardiomyopathies**	*ACTC1* (NM_005159.5)	*JUP* (NM_002230.4)	*RBM20* (NM_001134363.3)
	*ACTN2* (NM_001103.4)	*LAMP2* (NM_002294.3)	*SCN5A* (NM_000335.5)
	*BAG3* (NM_004281.4)	*LMNA* (NM_170707.4)	*TAZ* (NM_015472.6)
	*CSRP3* (NM_003476.5)	*MYBPC3* (NM_000256.3)	*TNNC1* (NM_003280.3)
	*DES* (NM_001927.4)	*MYH7* (NM_000257.4)	*TNNI3* (NM_000363.5)
	*DSC2* (NM_024422.6)	*MYL2* (NM_000432.4)	*TNNT2* (NM_001276345.2)
	*DSG2* (NM_001943.5)	*MYL3* (NM_000258.3)	*TPM1* (NM_001018005.2)
	*DSP* (NM_004415.4)	*NEXN* (NM_144573.4)	*TTN* (NM_001267550.1)
	*FLNC* (NM_014391.3)	*PKP2* (NM_001005242.3)	*TTR* (NM_000371.4)
	*GLA* (NM_000169.3)	*PLN* (NM_002667.5)	*VCL* (NM_014000.3)
**Genes associated with**	*CACNA1C* (NM_000719.7)	*CAV3* (NM_033337.3)	*KCNQ1* (NM_000218.3)
**primary arrhythmia**	*CACNB2* (NM_201596.3)	*HCN4* (NM_005477.3)	*RYR2* (NM_001035.3)
**disorders**	*CALM1* (NM_006888.6)	*KCNE1* (NM_000219.6)	*SCN5A* (NM_000335.5)
	*CALM2* (NM_001743.6)	*KCNE2* (NM_172201.2)	*TRDN* (NM_006073.4)
	*CALM3* (NM_005184.4)	*KCNH2* (NM_000238.4)	
	*CASQ2* (NM_001232.4)	*KCNJ2* (NM_000891.3)	

Gene selection was based on information reported by the GenCC database (https://thegencc.org/), including Definitive, Strong, and Moderate levels of evidence supporting gene-disease relationships.^7–9^

### CNV annotation

eVai software, powered by enGenome, has been applied for variant calling, annotation and prioritization. FASTQ files were uploaded to eVai (Expert Variant Interpreter) software version (eVai tertiary v3.2, eVai secondary analysis v1.6), which automatically follows a bioinformatics pipeline, including quality controls, alignment to the human reference genome, and variant calling. In this analysis, FASTQ files were aligned to the human reference genome GRCh37.

This software has been implemented for CNV analysis using a custom baseline. Specifically, 20 controls—10 males and 10 females—without any familial correlation has been selected by each laboratory, and 8 different baselines have been created according to the platform and the sequencing kit used. Furthermore, in a subset of 63 patients, variant detection and interpretation were additionally performed using independent software tools, including Manta Structural Variant Caller (version 1.6.0–3), VarSeq, and the GENEYX analysis platform, to improve analytical robustness and reduce platform-specific bias.

### Variant selection process

We developed a three-step workflow for CNV variant selection.

Step 1—Comprehensive Analysis:

All CNVs underwent an initial quality control (QC) assessment. Furthermore, selected variants were filtered based on their case count reported by eVai (Lab finding <2) and their consistency with the known gene-disease association. Finally, only the CNVs classified as Likely Pathogenic or Pathogenic according to the American College of Medical Genetics and Genomics (ACMG) and the Clinical Genome Resource (ClinGen) guidelines.^10^

Step 2—Visualization Analysis:

Putative CNVs identified in Step 1 were visually confirmed using the Integrative Genomics Viewer (IGV) tool to assess the consistency of read-depth signals.

Step 3—Validation Analysis:

All CNVs that passed Step 1 were subsequently validated using an orthogonal method, specifically the Multiplex Ligation-dependent Probe Amplification (MLPA) assay, to confirm their presence and exclude false positives.

### Orthogonal confirmation

CNV validation has been performed by MLPA using the commercial SALSA® MLPA® Probemix P200-B1 Reference-1 kit, SALSA MLPA Probemix P100 MYBPC3 kit and SALSA MLPA Probemix P168 ARVC-PKP2 kit (MRC-Holland, Amsterdam, Netherlands) following the manufacturer’s protocol. The raw electropherograms were analyzed with GeneMapper (Applied Biosystems) and Coffalyser.Net (v.240129.1959) software.

Sample results were compared with a set of reference controls to calculate a probe ratio indicative of copy number status. The deletion threshold was set at 0.50, and the duplication threshold at 1.30.

The list of the light custom probes has been reported in the (Supplementary material online, *Table S1*).

## Results

We analyzed 203 individuals using an *in silico* panel of 49 genes (*Table 1*). Males accounted for 64% of the patients, with a mean age of 49.7 years. Thirty-two percent of the patients (64/203) have a reported family history for ICC.

Following the workflow developed for the first-step analysis, which included only CNVs that passed quality filters, we identified 119 intragenic CNVs. Specifically, 87% of these were located in genes associated with cardiomyopathies, while the remaining 13% were associated with inherited Primary Arrhythmias. CNVs were filtered based on gene-disease relationships and the internal case count. This analysis identified three putative causative CNVs in three patients. In addition, two uncommon CNVs were detected that are not strictly located in genes with established gene-disease associations but were considered for further evaluation: one in a patient without previous causative variants, and one in a patient already carrying a putative causative CNV.

Specifically, 40% of the CNVs affect a single exon, whereas the remaining involve multi-exon regions, ranging from 4 to 11 exons. No discrepancies were observed across the different analysis software tools.

These CNVs are located in five different genes associated with ICCs: *MYBPC3* (*n* = 1), *NEXN* (*n* = 1), *DSG2* (*n* = 1), *CACNB2* (*n* = 1), *PKP2* (*n* = 1). Results from IGV analysis supported the presence of 4 CNVs (80%) out of the 5 initially selected in the first step. The remaining CNV was considered a false positive after visual inspection of the corresponding CRAM files.

More specifically, we confirmed variants in 3 patients with inherited cardiomyopathies. In particular, patient Cardio149 carries two heterozygous deletions of 26 110 bp and 10 985 bp, located in the *NEXN* gene and *DSG2* gene, respectively. Details of the CNVs are reported in *Table 2*.

**Table 2:** Characteristics of CNVs selected in the three-step analysis

Patient ID	Diagnosis	Family history	Gene	CNV Coordinates (Grch37)	Region	DEL/DUP	State	Second step Visualization analysis results	Third step Validation analysis results	SNV/InDels variants	Sequencing method
GM23-35591	HCM	Yes	*MYBPC3*	Chr11:47353422-47355573	Exon 28–34	DEL	Heterozygous	Confirmed	Confirmed and breakpoints definition	No	WES
Cardio149	DCM	Yes	*NEXN*	Chr1:78381792-78407901	Exon 2–12	DEL	Heterozygous	Confirmed	Not confirmed	No	Sequencing panel
CCM7	ACM	No	*PKP2*	Chr12:32949034-32949244	Exon 11	DEL	Heterozygous	Confirmed	Not confirmed	No	Sequencing panel
CMP18–23	BrS	Yes	*CACNB2*	Chr10:18439800-18439912	Exon 2	DEL	Heterozygous	Not confirmed	Not confirmed	No	WES
Cardio149	DCM	Yes	*DSG2*	Chr18:29104399-29115383	Exon 7–10	DEL	Heterozygous	Confirmed	Not confirmed	No	Sequencing panel

**Figure 1:**
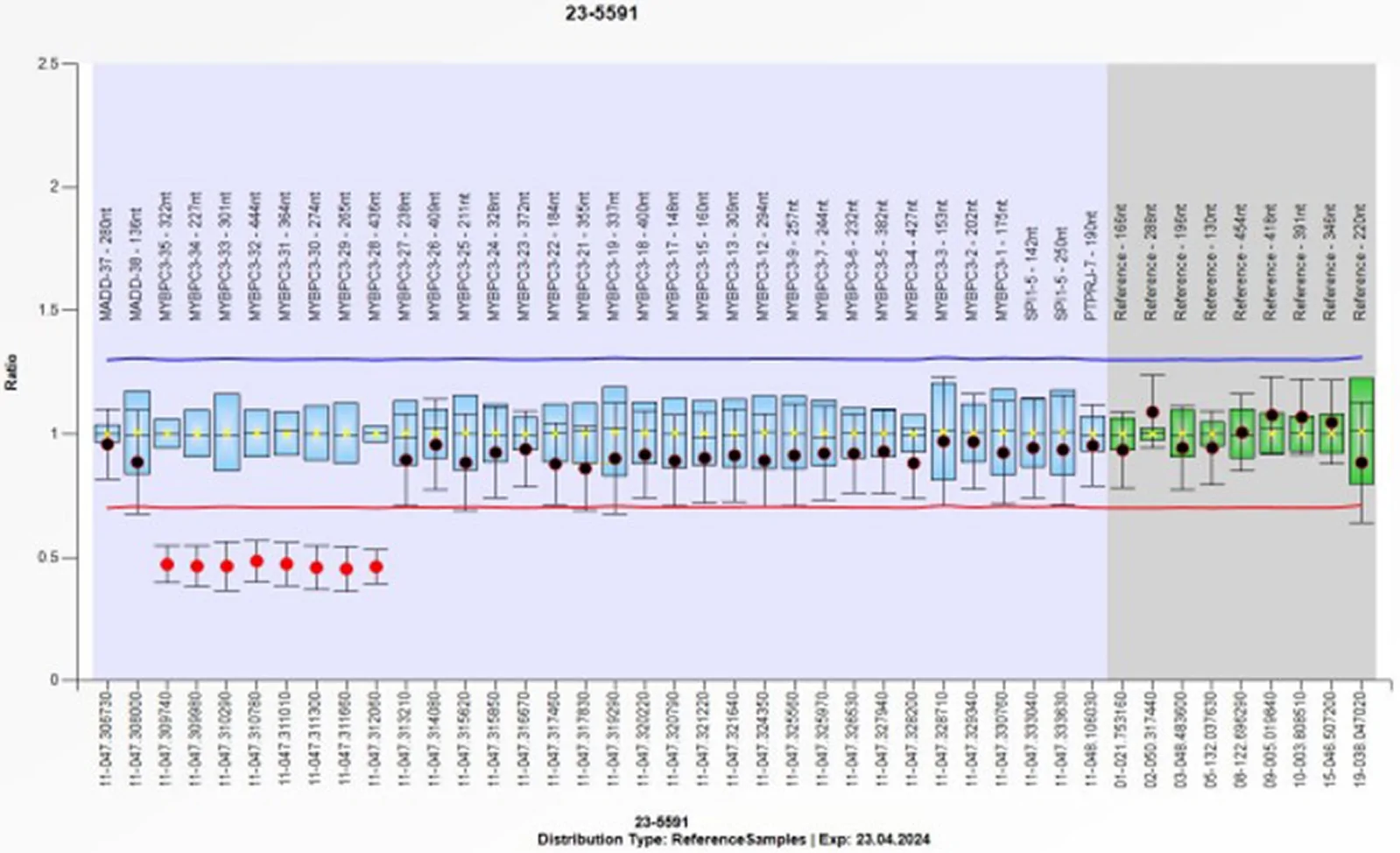
Results from MLPA analysis of MYBPC3 CNV. MLPA box plot showing copy number ratios across the *MYBPC3* gene. Exons 28–35 display reduced probe ratios (<0.50), consistent with a heterozygous deletion.

Finally, as the third step to confirm the accuracy of the selection applied in the second step of analysis, we performed MLPA validation on all CNVs detected. The study confirmed the presence of only one out of 5 (20%) CNVs, a deletion of 2152 bp in *MYBPC3* (NM_000256.3) gene involving exons from 28 to 34. In addition, MLPA analysis revealed the involvement of exon 35, not detected in the second step of analysis (*Figure 1*).

This variant was identified in a 26-year-old male patient clinically diagnosed with familial HCM. Cascade screening confirmed the presence of the variant also in the father (52 y.o.), clinically affected.

This CNV has been classified using the ACMG and Clinical Genome Resource (ClinGen) framework for the interpretation and reporting of CNVs.^10^

Evidence, including copy-number loss content (1A), both breakpoints within the same gene (2E), number of protein-coding RefSeq genes wholly or partially included in the copy number loss (3A), statistically significant increase amongst observations in cases compared with controls (4L), and CNV segregates with a consistent phenotype observed in the patient’s family (5D), confirmed the pathogenicity of the variant.

The remaining four CNVs selected in the second step were classified as false positives by MLPA, showing probe ratios close to 1.0, consistent with non-mutated sequences. Notably, the suspected *PKP2* alteration was re-evaluated using a second probe, further confirming the false-positive result. Overall, the cohort showed a molecular diagnostic yield of 0.5%.

### Strategy for incorporating CNV analysis according to patient-specific criteria

**Figure 2:**
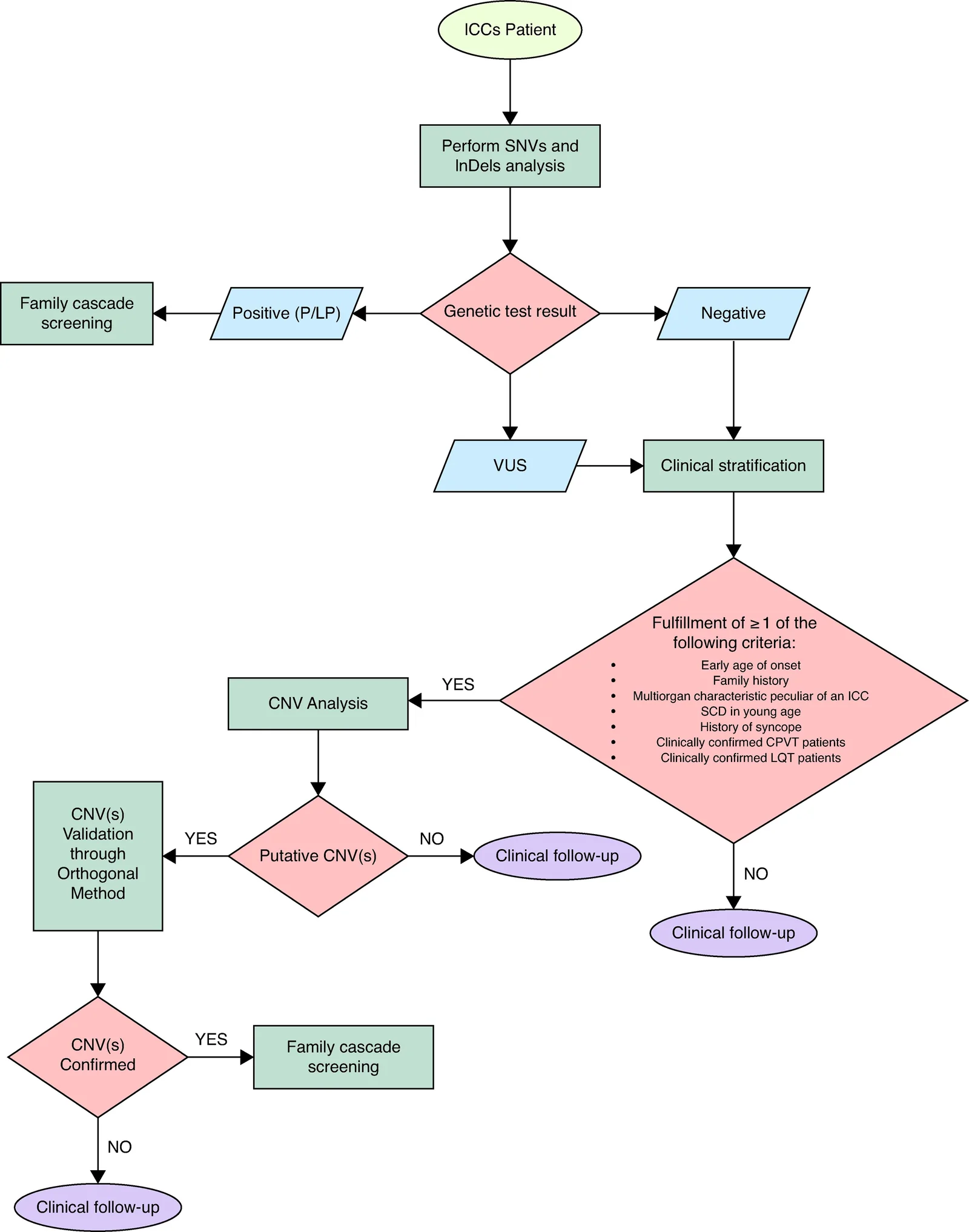
Algorithm to guide the selection of patients for CNV genetic testing.

Due to the limited number of CNVs detected in this cohort, we recommend implementing CNV analysis as a second-tier test, reserved for a selected subgroup of patients identified at high-risk according to the ESC guidelines.^11,12^ In *Figure 2*, an algorithm is proposed that may guide the selection of patients for this type of analysis.

This algorithm assumes that the patient presents features of recognizable ICCs, and analyses are pursued to evaluate only disease-related genes, excluding questionable genes. The first-tier test includes the SNVs and InDels genetic analysis. If no variants are identified, or if only VUS, classified according to the ACMG/AMP guidelines is detected, the test is considered inconclusive (right side of figure). In both cases, further clinical evaluation is warranted to determine whether a second-tier test should be pursued based on the clinical presentation. This second step includes an accurate re-evaluation of the clinical exams of the patients. Patients with an early age of onset, documented family history, multiorgan characteristic peculiar to an ICC (e.g, deafness, keratoderma, woolly hair) or SCD at a young age, and history of syncope are selected to undergo the second-tier analysis. Furthermore, in light of the high prevalence of CNVs in specific arrhythmogenic disorders, second-tier analysis is strongly recommended for patients with a confirmed clinical diagnosis of Long QT Syndrome (LQTS) or CPVT, where structural rearrangements in genes such as *KCNQ1* or *RYR2* may be missed by standard NGS pipelines.^13–15^

If a CNV is detected within a known, clinically relevant region or gene, or if the CNV is in the genomic backbone and meets recommended size and gene content guidelines, then the result is considered a pathogenic CNV. In this case, confirmation with an orthogonal method is mandatory.

In case of pathogenic or likely pathogenic SNV, InDels, or CNV, cascade screening of the family members is highly recommended.

This algorithm enables to maximize the diagnostic yield specifically in high-risk subgroups, where the pre-test probability of detecting clinically relevant CNVs is significantly higher, thereby improving the overall efficiency and clinical utility of genetic testing.

## Discussion

The objective of this study was to assess the utility of implementing copy number variant (CNV) analysis within routine genetic screening for ICCs. By examining an unselected multicentre cohort with inconclusive prior testing, we observed a relatively low diagnostic yield of 0.50%.

Several studies have investigated the contribution of CNVs in ICCs, including both cardiomyopathies and channelopathies, using different sequencing approaches and analytical pipelines. In cardiomyopathies, CNVs account for a highly variable diagnostic yield, largely influenced by cohort selection and gene panel composition. The most comprehensive study investigating the prevalence of intragenic CNV in Mendelian disorders, has reported a CNV detection rates ranging from 4.7% to 35%, with an overall rate of ∼4.7% in cardiovascular disorders, varying from 0% to 16.7% depending on the gene panel analyzed.^3^ However, studies conducted in unselected cardiomyopathy cohorts have consistently reported a low prevalence of pathogenic CNVs, generally below 1%.^2,16,17^ For example, Ceyhan-Birsoy *et al*.^17^ described a CNV detection rate of 0.28% in a large cohort of patients with inherited cardiomyopathies, supporting a limited but measurable contribution of CNVs in these conditions^17^ (see Supplementary material online, *Table S2*).

Higher diagnostic yields have been reported in highly selected or phenotype-enriched cohorts, particularly in studies focused on definitive, disease-associated genes such as *MYBPC3* and *MYH7* in HCM, where CNV detection rates range from 1% to 1.4%^18,19^ (see Supplementary material online, *Table S2*). These findings suggest that increased diagnostic yield is mainly driven by cohort specificity and careful gene selection. Therefore, CNV analysis should preferably be restricted to genes with definitive disease association, as excessively broad panels may increase the detection of variants with uncertain or limited clinical relevance.

Similarly, studies focusing on channelopathies have reported more heterogeneous results, with a wider range of CNV detection rates. A study by Priori SG *et al*. identified CNVs in 12 out of 88 probands with Long QT syndrome, corresponding to a prevalence of 14% in *KCNQ1* and *KCNH2*, with 33% of carriers presenting with syncope prior to diagnosis.^13^ This relatively high yield likely reflects the inclusion of a clinically enriched cohort and the targeted analysis of major disease genes. In CPVT, studies in highly selected patients—often with severe phenotypes, including a history of fatal arrhythmias and negative prior genetic testing—have reported CNV detection rates of 6% to 8.3% in *RYR2*.^14,15^ These observations are consistent with the high overall diagnostic yield (∼80%) and the well-established molecular basis of these channelopathies (see Supplementary material online, *Table S2*).

Overall, the available literature indicates that CNV detection rates in ICCs are highly dependent on cohort characteristics, phenotype definition, and methodological approaches, with consistently lower yields observed in unselected populations. In this context, our findings of a low CNV detection rate (0.50%) are in line with previous reports, as highlighted in Supplementary material online, *Table S2*. Furthermore, it is worth noting that the diagnostic yield of CNVs often differs between sequencing strategies; while WES offers a broader view of the exome, targeted Gene Panels frequently demonstrate higher sensitivity for CNVs within specific loci due to superior depth of coverage and specialized bioinformatics pipelines.^20^ Importantly, our proposed algorithm is not based solely on the single positive case identified in this study, but is supported by evidence suggesting that CNV analysis is most informative in selected high-risk subgroups rather than in unselected cohorts.

Based on these considerations, we propose including CNV analysis as a second-tier genetic test in selected patients. This approach is supported not only by the relatively low diagnostic yield in unselected populations but also by the technical and interpretative challenges associated with CNV detection, which increase both turnaround time and overall costs. Validation using orthogonal methods, such as array comparative genomic hybridization (aCGH),^21^ quantitative PCR (qPCR),^22^ or MLPA,^23^ remains mandatory to confirm CNVs and accurately define breakpoints. Although these methods are resource-intensive, they are essential to ensure the reliability of CNV interpretation in a diagnostic setting.

We therefore propose the clinical algorithm shown in *Figure 2* to guide the implementation of CNV analysis in routine genetic testing. This approach aims to maximize diagnostic yield in high-risk subgroups, where the pre-test probability of detecting clinically relevant CNVs is higher, thereby improving the overall efficiency and clinical utility of genetic testing. Current European Society of Cardiology (ESC) guidelines do not specify which classes of genetic variants should be prioritized during diagnostic testing.^11,12^ The present data demonstrate the potential clinical utility of CNV analysis in selected high-risk subgroups suggesting that future guideline updates should consider incorporating these data to refine recommendations on comprehensive genetic testing strategies.

## Conclusions

Genetic testing has become a powerful tool in molecular cardiology. However, the diagnostic yield varies widely across different cardiac diseases, ranging from 20% to 80%. Investigating the role of CNVs in the context of ICCs may help to decrease this variability, improving diagnostic rates in selected cases. Our work reported the limited contribution of this SV in an unselected population of ICC. Recommendations for the implementation of this analysis in selected patients have been suggested.

## Supplementary Material

euag150_Supplementary_Data

## Data Availability

The data underlying this article are available in the article and in its online Supplementary material.
