# Supplementary material for: The evaluation of copy number variants in an unselected population of patients with inherited cardiac conditions: the INTERACTION study
Source: Europace. 2026 Jun 18;28(7):euag150. doi: 10.1093/europace/euag150 (PMC13331274; doi:10.1093/europace/euag150)
Supplement: euag150_Supplementary_Data [file euag150_supplementary_data.zip › Supplementary_Materials_S1.docx]

**Supplementary Material**

Targeted NGS was performed by six centers using the MiSeq System (Illumina, San Diego, CA, USA) following the manufacturer’s protocol. Sequencing libraries were prepared with the TruSight Cardio Sequencing Kit, which targets 174 genes associated with inherited cardiac conditions. Two centers — Centro Cardiologico Monzino and Ospedale Maggiore della Carità di Novara — used the TruSight panel, while the remaining four laboratories used different custom cardiovascular gene panels.

WES was performed in two laboratories: Ospedale Bambino Gesù di Roma used the NovaSeq System (Illumina, San Diego, CA, USA), and Ospedale Maggiore Policlinico di Milano used the NextSeq 550 System (Illumina, San Diego, CA, USA). Library preparation for WES was carried out according to the manufacturer’s instructions.
