# Supplementary material for: The evaluation of copy number variants in an unselected population of patients with inherited cardiac conditions: the INTERACTION study
Source: Europace. 2026 Jun 18;28(7):euag150. doi: 10.1093/europace/euag150 (PMC13331274; doi:10.1093/europace/euag150)
Supplement: euag150_Supplementary_Data [file euag150_supplementary_data.zip › Table_S1.docx]

| **Probes** | **Sequence (5’-3’)** |
| --- | --- |
| Cardio149_NEXN_LPO | GGGTTCCCTAAGGGTTGGAGTGGAAAGGTCATATTTAGGAGTTCATAATTAAT |
| Cardio149_NEXN_RPO | AACATAATGGCAAGATGTAAAACAAGAAGAGATATCTAGATTGGATCTTGCTGGCAC |
| Cardio149_DSG2_LPO | GGGTTCCCTAAGGGTTGGACGTATTTTGGATGTCAATGACAATATACCTG |
| Cardio149_DSG2_RPO | TAGTAGAAAATAAAGTGGTAACTATTATTCTTCTAGATTGGATCTTGCTGGCAC |
| CMP18-23_CACNB2_LPO | GGGTTCCCTAAGGGTTGGAGAAAAACAGATTTAAAGGATCTGATGGAAGCACGTCA |
| CMP18-23_CACNB2_RPO | TCTGATACTACCTCAAATAGTTTTGTTCGCCAGGTAATCTAGATTGGATCTTGCTGGCAC |
| CCM7_PKP2_LPO | GGGTTCCCTAAGGGTTGGAGGGGAAAGGGAGGCAGCTGACGGGCAGAACT |
| CCM7_PKP2_RPO | GAAGGACTTACGCATCGCCTGCACTAATGGCTCTAGATTGGATCTTGCTGGCAC |

**Table** **S1**
