# Supplementary material for: The evaluation of copy number variants in an unselected population of patients with inherited cardiac conditions: the INTERACTION study
Source: Europace. 2026 Jun 18;28(7):euag150. doi: 10.1093/europace/euag150 (PMC13331274; doi:10.1093/europace/euag150)
Supplement: euag150_Supplementary_Data [file euag150_supplementary_data.zip › Table_S2.docx]

**Table S2**

| **Reference** | **# Ref** | **Phenotype** | **Cohort size (n)** | **Cohort type** | **CNV detection method** | **Testing strategy** | **Yield (%)** | **CNV type** | **Discussion points** |
| --- | --- | --- | --- | --- | --- | --- | --- | --- | --- |
| Truty et al., 2019 | 3 | ICCs | 143,515 | individuals referred for genetic testing | NGS + MLPA | Multi-panels | 4.7% | Deletions/  Duplications | Wide variability in diagnostic yield across cardio gene panels (0–16.7%). Unselected population. |
| de Uña-Iglesias et al., 2024 | 2 | Cardiomyopathies | 11,647 | individuals referred for genetic testing | NGS + MLPA/ddPCR | Panel (170 genes) | 0.8% | Deletions/  Duplications | Wide variability in diagnostic yield across phenotype (0,29% HCM–1,88% ACM). Unselected population. |
| Heliö et al.,2023 | 16 | DCM | 2,088 | individuals referred for genetic testing | NGS + qPCR + ddPCR | Multi-panels (23–316 genes ICC genes) | 0.6% | Deletions/  Duplications | 172 patients were genotype-negative for SNVs. Different panels were used to define the yield. |
| Ceyhan-Birsoy et al.,2016 | 17 | Cardiomyopathies | 1,425 | individuals referred for genetic testing | NGS + ddPCR | Multi-panel (≤46 genes per panel) | 0.63% | Deletions/  Duplications | Wide variability in diagnostic yield across cardio gene panels (0,4%HCM–1,9% LVNC). |
| Chanavat et al., 2012 | 18 | HCM | 100 | SNV Genotype-negative cohort | MLPA + LR-PCR | *MYBPC3* | 1% | Deletion | Selected cohort, but the analysis is restricted to a single gene, limiting its scope. |
| Pezzoli et al., 2012 | 19 | HCM | 72 | SNV Genotype-negative cohort | MLPA + LR-PCR | *MYBPC3, TNNT2* | 1.4% | Deletion | Selected cohort, but the analysis is restricted to 2 of the 9 sarcomeric genes, limiting its scope. |
| Trancuccio et al., 2025 | 13 | Long QT syndrome | 88 | SNV Genotype-negative cohort | NGS + MLPA | Panel (*KCNQ1*, *KCNH2*) | 14% | Deletions/  Duplications | Selected cohort with targeted analysis of 2 of the 3 major disease genes. |
| Marjamaa et al., 2009 | 14 | CPVT | 33 | individuals referred for genetic testing | MLPA + Sanger sequencing | *RYR2* | 6% | Deletions | Recurrent RYR2 exon 3 deletions in unrelated CPVT families support the relevance of the CNV analysis in these patients. |
| Ohno et al.,2014 | 15 | CPVT | 24 | SNV Genotype-negative cohort | MLPA + LR-PCR | *RYR2* | 8.3% | Deletions | Highly selected patients with prior negative genetic testing and targeted CNV analysis in the main gene. |

SNV=Single Nucleotide Variant

qPCR= quantitative Polymerase Chain Reaction

ddPCR=digital droplet Polymerase Chain Reaction

LR-PCR=long range Polymerase Chain Reaction
